# Applying landscape genomic tools to forest management and restoration of Hawaiian koa (*Acacia koa*) in a changing environment

**DOI:** 10.1111/eva.12534

**Published:** 2017-09-06

**Authors:** Paul F. Gugger, Christina T. Liang, Victoria L. Sork, Paul Hodgskiss, Jessica W. Wright

**Affiliations:** ^1^ Ecology and Evolutionary Biology University of California Los Angeles CA USA; ^2^ Appalachian Laboratory University of Maryland Center for Environmental Science Frostburg MD USA; ^3^ USDA Forest Service Pacific Southwest Research Station Hilo HI USA; ^4^ Institute of the Environment and Sustainability University of California Los Angeles Los Angeles CA USA; ^5^ USDA Forest Service Pacific Southwest Research Station Davis CA USA

**Keywords:** *Acacia koa*, climate change, generalized dissimilarity modeling, genotype–environment association, genotyping by sequencing, gradient forest, Hawaii, landscape genomics

## Abstract

Identifying and quantifying the importance of environmental variables in structuring population genetic variation can help inform management decisions for conservation, restoration, or reforestation purposes, in both current and future environmental conditions. Landscape genomics offers a powerful approach for understanding the environmental factors that currently associate with genetic variation, and given those associations, where populations may be most vulnerable under future environmental change. Here, we applied genotyping by sequencing to generate over 11,000 single nucleotide polymorphisms from 311 trees and then used nonlinear, multivariate environmental association methods to examine spatial genetic structure and its association with environmental variation in an ecologically and economically important tree species endemic to Hawaii, *Acacia koa*. Admixture and principal components analyses showed that trees from different islands are genetically distinct in general, with the exception of some genotypes that match other islands, likely as the result of recent translocations. Gradient forest and generalized dissimilarity models both revealed a strong association between genetic structure and mean annual rainfall. Utilizing a model for projected future climate on the island of Hawaii, we show that predicted changes in rainfall patterns may result in genetic offset, such that trees no longer may be genetically matched to their environment. These findings indicate that knowledge of current and future rainfall gradients can provide valuable information for the conservation of existing populations and also help refine seed transfer guidelines for reforestation or replanting of koa throughout the state.

## 1 INTRODUCTION

Identifying and quantifying the importance of environmental variables in structuring population genetic variation can inform management decisions for conservation, restoration, or reforestation purposes, in both current and future environmental conditions. For example, knowledge about the association between genotype and environment is important for selecting proper seeds for planting when tree populations are locally adapted (Aitken & Whitlock, 2013; Sork et al., 2013), as is generally the case (Rehfeldt, Ying, Spittlehouse, & Hamilton, 1999; Savolainen, Pyhajarvi, & Knurr, 2007). As the environment changes, nonlocal seed sources may be increasingly considered based on their match to the novel environment. Therefore, seed transfer guidelines can benefit from knowledge of the factors structuring genetic variation on the landscape and how they may change in the future.

Spatial genetic structure can arise due to restricted dispersal and geographic barriers to gene flow (Avise, 2000), as well as environmental factors that shape genetic variation on the landscape by influencing demographic processes (e.g., via phenology) or imposing natural selection that leads to local adaptation (collectively, “isolation by environment”; Sexton, Hangartner, & Hoffmann, 2014; Wang & Bradburd, 2014). Many conservation efforts rely on delineating distinct populations for management but often ignore the continuous nature of variation on the landscape and its potential relationship with local adaptation or other processes that lead to genotype–environment associations (Frankham, 2010; Rodríguez‐Quilón et al., 2016). However, it is important to understand both geographic and environmental factors considering the potential for complex relationships on the landscape.

A landscape genomics approach, utilizing large numbers of genetic loci, offers a powerful means of detecting subtle genetic variation along the landscape in relation to geographic and environmental variables (Sork et al., 2013). When coupled with emerging analytical methods to explore nonlinear genotype–environment associations in multivariate space, this approach can enhance our ability to explain and quantify modern patterns and then project them into the future to identify vulnerable or resilient populations along the landscape (Fitzpatrick & Keller, 2015; Holliday et al., 2016). Two promising models are gradient forest (Ellis, Smith, & Pitcher, 2012) and generalized dissimilarity modeling (Ferrier, Manion, Elith, & Richards, 2007), which were first applied to community ecological data sets and have recently been advocated for landscape genomics (Fitzpatrick & Keller, 2015). Each can be used to quantify the role of particular environmental and spatial variables in structuring genetic variation and describe potentially nonlinear rates of change along these gradients, thus testing for isolation by environment in a more realistic and informative way than most linear models. Furthermore, they can be considered complementary to each other because they approach genotype–environment associations in very different ways: Gradient forest is regression tree‐based, whereas generalized dissimilarity modeling is distance‐based.

The Hawaiian Islands provide an excellent system to apply landscape genomics for current conditions as well as for future scenarios, as the archipelago encompasses a wide range of geographic and environmental variation with sharp gradients within a relatively small area within and between islands (Vitousek, 1995). The geologic history of the archipelago has a direct impact on the distribution of genetic variation across the islands, and the extant islands range in age from 5.1‐million‐year‐old Kauai to the still‐growing island of Hawaii. Colonization of the land by plants likewise ranges from millions of years to newly colonized (Price & Clague, 2002), and the differences in the geological age of rock substrates on the island of Hawaii can impact plant communities (Kitayama, Mueller‐Dombois, & Vitousek, 1995). The remoteness of the Hawaiian Islands has resulted generally in a species‐poor but unique biota, with a large number of endemic species including a few examples of dramatic adaptive radiation (Carr & Kyhos, 1981; Craddock & Kambysellis, 1997). Many of the endemic species are of conservation concern, making landscape genomic investigations timely and highly applicable for management purposes.

One such case is for the ecologically, economically, and culturally important species, *Acacia koa* A. Gray (koa). Koa is an endemic outcrossing leguminous hardwood tree that has been under threat due to land use changes, logging, and the introduced fungal pathogen *Fusarium oxysporum* f. sp. *koae* (Baker, Scowcroft, & Ewel, 2009). *A*. *koa* is one of two dominant canopy species, along with *Metrosideros polymorpha* (‘ō’hia), in native Hawaiian forests. It is distributed on all the main Hawaiian Islands, except Niihau and Kahoolawe, and has the greatest densities on Hawaii, Maui, Oahu, and Kauai (Wagner, Herbst, & Sohmer, 1999). It is found in a broad range of environments from dry to semi‐saturated rain forests, and from sea level to more than 2,000 m in elevation. The largest extant populations of koa are found on the island of Hawaii between 1,000 and 2,000 m (Baker et al., 2009). Koa exhibits phenotypic diversity with two forms that are generally recognizable based on morphology. A shorter form with narrower phyllodes and longitudinally arranged seeds in pods is referred to sometimes as *A. koaia* and is found in drier areas of Hawaii, Maui, Lanai, and Molokai (Wagner et al., 1999). A taller form with broader phyllodes and transversely arranged seeds in pods is referred to generally as *A*. *koa*. Based on molecular analysis using the nuclear ITS, chloroplast *trn*K introns, and microsatellite markers, findings from Adamski, Dudley, Morden, and Borthakur (2012) support previous recommendations that recognize the morphological variations at the subspecific level within *A*. *koa*. Genetically based morphological and growth differences are also apparent among *A. koa* from the island of Hawaii and other islands (Daehler, Yorkston, Sun, & Dudley, 1999; Shi, 2003; Sun, 1996). Although unusual and difficult to explain, *A. koa* may be paraphyletic with its closest relative, *A. heterophylla*, which itself is monophyletic and is endemic to Réunion Island in the Indian Ocean over 16,000 km away from Hawaii (Le Roux et al., 2014). Seed transfer guidelines for ecological restoration and agroforestry have been proposed based on ecological zones among and within islands as well as preliminary genetic analyses (Dudley et al., 2017), but would benefit from insights from landscape genomic approaches that integrate environmental and genetic components.

To characterize patterns of genetic variation in *A. koa* across Hawaii and identify and quantify key climate variables associated with that genetic variation, we analyzed allele frequencies of single nucleotide polymorphisms (SNPs) in relation to spatial and environmental variables in two predictive nonlinear modeling frameworks: gradient forest and generalized dissimilarity modeling. Our study objectives were to (i) examine genomewide, biogeographic patterns of genetic differentiation in koa across the Hawaiian Islands, (ii) identify the most important geographic and environmental variables structuring genetic variation within the island of Hawaii, and (iii) assess whether koa populations might be vulnerable to potential future environmental changes by applying predicted future climate model data available for the island of Hawaii.

## 2 METHODS

### 2.1 Sampling

We sampled 311 trees across the geographic, elevational, and climatic range of koa on the islands of Hawaii, Maui, Oahu, and Kauai, including six putative *A. koaia* samples from the island of Hawaii, from November 2012 to September 2013 (Table S1).

### 2.2 Genotyping by sequencing

Total genomic DNA was extracted from frozen tissue using the NucleoSpin kit (Macherey‐Nagel, Bethlehem, PA, USA). DNA was prepared for sequencing using an efficient restriction‐enzyme‐based approach commonly known as genotyping by sequencing (GBS) (Elshire et al., 2011). Briefly, DNA was digested with the *Ape*KI restriction enzyme, common and unique barcoded adapters with overhangs complementary to the cut site were ligated to each sample, 48 samples were pooled in equimolar ratios, and the pooled library was PCR‐amplified and sent for Illumina sequencing. We largely followed the original protocol of Elshire et al. (2011), including using the same adapter concentration. However, a different set of longer barcode sequences were used (Table S2), all steps were performed manually rather than robotically, and we made a few changes to optimize the protocol for *Acacia*, consistent with our experience using similar approaches with *Quercus* spp. (P.F. Gugger & V.L. Sork, unpublished). For example, adapters were added during the ligation step rather than added to the empty plate and dried down prior to digestion; AMPure XP bead‐based size selection/purification steps were added after the ligation step and repeated after the PCR step to ensure a consistent distribution of fragment sizes between 200 and 500 bp among all preps; and we reduced the number PCR cycles to 16 from 18. Final libraries were checked for the proper size distribution on an Agilent BioAnalyzer (Santa Clara, CA) with the High Sensitivity DNA assay and quantified using a Qubit fluorometer (Waltham, MA). Samples were sent to the UCLA Broad Stem Cell Research Center for single‐end, 100‐bp sequencing on an Illumina HiSeq2000 v3 (San Diego, CA).

### 2.3 Single nucleotide polymorphism calling

SNPs were identified using stacks 1.35 (Catchen, Amores, Hohenlohe, Cresko, & Postlethwait, 2011; Catchen, Hohenlohe, Bassham, Amores, & Cresko, 2013). Raw Illumina data in FASTQ format were quality‐filtered and demultiplexed using process_radtags, which removed adapter sequence with up to two mismatches (‐‐adapter_mm), recovered reads whose barcodes had up to one mismatch to the expected barcodes (‐r), removed any read with an uncalled base (‐c), discarded low‐quality reads as defined by default settings (‐q), and trimmed all reads to 87 bases (‐t). Parameters for subsequent steps were optimized based on three samples, one from each of three Hawaiian Islands, that were replicated across four library preparations and lanes of Illumina sequencing. We ran stacks 1.21 repeatedly on these replicates with a variety of parameter values by varying one at a time in each run. Specifically, we evaluated ‐m 3–5, ‐M 1–3, ‐n 1–3, ‐‐max_locus_stacks 3–5, and ‐‐bound_high 0.05 or 0.1 (Figs S1 and S2). The “optimal” parameter values were those that minimized differences among replicates as inferred from ordinations of the resulting SNPs. This procedure minimizes the potential SNP and genotype calling error in the spirit of other optimization procedures (Mastretta‐Yanes et al., 2015). The ordinations included multidimensional scaling based on Hamming distance (Fig. S1) and principal components analysis (Fig. S2), which were performed in PLINK 1.90b2n (Chang et al., 2015). We concluded that the “optimal” values for our data were ustacks parameters ‐m 4 (minimum stack depth to retain locus in an individual) and ‐M 1 (maximum distance between stacks to combine them into a locus) and cstacks parameter ‐n 1 (number of mismatches allowed to combine locus among samples when creating catalog). In comparison with ‐m 4, ‐m 3 also produced similarly small differences among replicates (i.e., low error) when in combination with ‐M 1 ‐n 1 but yielded nearly twice as many total SNPs; thus, we preferred –m 3 to –m 4. Little difference was observed among ‐‐bound_high and ‐‐max_locus_stacks alternatives; thus, values of 0.05 and 3 were chosen, respectively. Regardless of the parameter values selected, summary statistics of diversity, such as heterozygosity (0.22 < *H *<* *0.30) and nucleotide diversity (0.0010 < π < 0.0017), did not vary substantially (Table S3). Quality filters were applied to the pipeline to retain a high‐confidence subset of SNPs; with rxstacks, we removed SNPs with ln*L* < −30.0 (–‐lnl_lim), proportion of “confounded” loci > 0.25 (‐‐conf_filter), and nonbiological haplotypes (‐‐prune_haplo). In each pass of the stacks pipeline through cstacks, the catalog (a reference set of sequences) was built using a geographically representative subset of 14 samples, as is recommended. Only one SNP per “stack” was retained, and SNPs with >30% missing data across all samples (*n *=* *311), ln*L* < −30 (populations –r 0.7 –‐lnl_lim ‐30 –‐write_single_snp), or minor allele frequency <0.05 were discarded. Another set of SNPs was generated with the same filters considering only samples from the island of Hawaii (*n *=* *207, or *n *=* *201 without *A. koaia*).

### 2.4 Ploidy

Koa is tetraploid and does not exhibit variation in ploidy (Atchison, 1948; Carr, 1978; Hipkins, 2004; Shi, 2003), but it is unclear whether it is an autotetraploid or allotetraploid. Some authors have speculated that koa is an allotetraploid with disomic inheritance based on inconclusive evidence (Shi, 2003; Shi & Brewbaker, 2005). In support of this view, isozyme data (Conkle, 1996) often show more than two alleles at a locus within an individual. In contrast, others have argued that *A. koa* is autotetraploid because its closest relative, *A*. *heterophylla,* which is phylogenetically nested within *A. koa*, is a putative autotetraploid formed from another diploid *Acacia* species (Le Roux et al., 2014).

stacks is designed for diploid species, but can call SNPs in polyploids if the error model is adjusted to consider the possibility that reads supporting each allele in a heterozygote may deviate substantially from 50:50. We did so by setting ‐‐bound_high (the maximum sequencing error rate) to 0.05 as indicated above. Nonetheless, the resulting genotype calls are “coerced” to appear diploid. If koa is an allotetraploid (amphidiploid) formed from two species, then this coercion may be appropriate because divergent homeologous loci are likely to be separated into separate loci in the stacks pipeline under our optimization procedure. If koa is autotetraploid, then we expect allele frequencies to be biased toward intermediate values because allelic dosages of 0.25 and 0.75 will be coerced to 0.5. We expect this coercion to occur equally in both directions (from 0.75 to 0.5 and from 0.25 to 0.5); thus, the primary effect would be to bin all heterozygotes as 0.5. This bias toward intermediate allele frequencies in individuals would not bias the association of these allele frequencies with environmental variables on the landscape (see description of methods below), but the binning may reduce power to detect such associations. Therefore, we are confident that our pipeline is unlikely to lead to erroneous conclusions from downstream analyses.

To explore the possibilities that koa is autotetraploid or that it is allotetraploid and our pipeline does not split homeologous loci, we generated histograms of the fraction of reads supporting each allele for each locus with at least 60 × coverage for each individual with at least 1,000 such loci. If either of these issues is present, we expect to find peaks at 0.25, 0.5, and 0.75, rather than just 0.5 (Arnold, Kim, & Bomblies, 2015).

### 2.5 Genetic diversity and structure

Mean expected heterozygosity (*H*) and nucleotide diversity (π) were estimated for each island using stacks. Genetic structure was estimated with admixture 1.3.0 (Alexander, Novembre, & Lange, 2009) considering *K *= {1, 2, 3, …, 8} clusters across all samples/islands and within only the island of Hawaii (*n *=* *207). The “optimal” number of clusters was chosen based on the *K* with the lowest cross‐validation error, as recommended by the developers. For the data set containing all samples, pairwise *F*_ST_ was estimated among clusters using admixture and compared to estimates among the four Hawaiian Islands as calculated in PLINK 1.90b3.29. These *F*_ST_ estimates are useful for relative comparisons and are unlikely to be biased substantially using diploid rather than tetraploid SNP calls, because the effect allele frequency binning averages out over the large number of SNPs. In addition, we performed principal components analyses using PLINK to provide a means for visualizing continuous changes in genetic structure complementary to the discrete clustering approach of admixture.

### 2.6 Environmental and spatial associations with genetic variation

To quantify the contribution of environmental and spatial variables in structuring genetic variation, we performed two types of nonlinear analyses, gradient forest (GF) and generalized dissimilarity modeling (GDM), focusing on samples of *A. koa* within the island of Hawaii (*n *=* *201). These approaches are complementary, as GF is a regression tree approach and GDM is a distance‐based approach. All analyses were performed on an individual basis, rather than arbitrary “population” groupings, which are not straightforward with our sampling design and observed patterns of genetic structure and admixture (see Results). In addition to spatial data based on GPS coordinates for each tree, we used the following environmental variables as predictors for both analyses: log_10_ of the mean estimated volcanic rock substrate age from a U.S. Geological Survey geologic map (Sherrod, Sinton, Watkins, & Brunt, 2007), mean annual rainfall (mm) from the 2011 Rainfall Atlas of Hawaii (Giambelluca et al., 2013), mean minimum temperature (°C) from the 2014 Climate of Hawaii project (Giambelluca et al., 2014), and isothermality (mean diurnal range ÷ mean annual range), temperature seasonality (standard deviation × 100), and rainfall seasonality (coefficient of variation) calculated from the above climate data sources in ArcGIS 10.0 (ESRI, Redlands, CA, USA) (Fig. S3). These specific environmental variables were chosen as a representative set that reflects factors expected to influence koa and that generally have correlations (|*r|*) < .8 with each other. Mean minimum temperature and mean annual rainfall are highly correlated (*r *>* *.95) with other mean temperature and rainfall variables, respectively, that were not included (Table S4).

Gradient forest analysis was implemented in “gradientForest” (http://gradientforest.r-forge.r-project.org/) in R 3.1.2 (R Development Core Team). GF is a nonparametric, machine‐learning regression tree approach (Ellis et al., 2012) that allows for exploration of nonlinear associations of spatial, environmental, and allelic variables. The approach partitions the allele frequency data at split values along the environmental gradients. Split importance, a measure of the amount of variation explained, is high in positions along the gradient where allelic change is large. Moving along the gradient, the split importance values are summed cumulatively to produce a steplike function for allele frequency change along the environmental gradient. For this analysis, we used the same SNP data in the form of allelic variables as above, except that missing data were retained rather than imputed. Spatial variables were defined using principal coordinates of neighborhood matrices (PCNMs), also known as Moran's eigenvector maps (MEM), based on the geographic coordinates in decimal degrees using the pcnm function in “vegan” (Oksanen et al., 2016). PCNMs are a set of orthonormal variables calculated through eigenvalue decomposition of a spatial weighting matrix, in our case, based on *x*–*y*‐coordinates (Dray, Legendre, & Peres‐Neto, 2006). We retained half of the PCNM variables with positive eigenvalues (*n *=* *26), as has been suggested in similar contexts (Fitzpatrick & Keller, 2015; Manel et al., 2012).

Generalized dissimilarity modeling (Ferrier et al., 2007), which is a distance‐based, nonlinear extension of matrix regression, was implemented in the R package “gdm” 1.2.3 (Manion, Lisk, Ferrier, Nieto‐Lugilde, & Fitzpatrick, 2016). GDM can account for nonlinear relationships between genetic distance and environmental and geographic distance, as well as variation in the rate of allelic compositional change along environmental gradients by fitting splines (Fitzpatrick & Keller, 2015). Spline shape describes the allelic compositional change along the environmental gradient, while spline height describes the importance of the particular environmental variable. Genetic distances among individuals were calculated as Euclidean distance based on allelic variables. Geographic distance was accounted for in the GDM based on Euclidean distance among coordinates (geo=T).

### 2.7 Mapping genetic offset under future climate

To investigate how these analyses can be used for management purposes, we identified parts of the *A. koa* distribution that might be vulnerable to anticipated climate change by estimating the expected “genetic offset” between one future climate scenario and the current landscape patterns (as estimated above) following Fitzpatrick and Keller (2015). Genetic offset is a measure of the magnitude of genetic change required between present and future climate to maintain the currently observed relationship between genetic and environmental variation. In these analyses, we excluded volcanic rock substrate age because it was not found to be a significant contributor to genetic patterns on the landscape (see Results) and because future rock age is directly linearly related to current rock age. As an estimate of future climate in Hawaii, we chose to use IPCC5 CMIP5 data at 30 arcsec resolution from the CESM1‐CAM5‐1‐FV2 global circulation model under the Representative Concentration Pathway 4.5 greenhouse gas emissions scenario for the year 2070 (average of 2061–2080) (Fig. S4). These data offer a moderate, representative scenario for demonstrative purposes, as data optimized specifically for Hawaii are not all available for our analyses.

For projecting GF results, we first used the GF predict function to predict genetic variation across all grid cells on the island of Hawaii. We then mapped the resulting predictions across the landscape masked by the expected distribution of *A. koa*/*A. koaia* (J.P. Price et al., 2012) using principal components of the predictions to generate a red–green–blue color scale according to the first three axes. Genetic offset under the future climate scenario was then estimated by first using the GF predict function with the future climate data and then estimating Euclidean distance between current predictions and future predictions weighted by variable importance for each grid cell on the landscape. A similar procedure was followed for GDM results using the gdm.transform and GDM predict functions with principal components to predict and map current genotype–environment relationships onto the landscape. The GDM predict function was then used with both current and future climate data rasters and time=T to estimate genetic offset in a single step.

## 3 RESULTS

### 3.1 Single nucleotide polymorphisms and ploidy

We identified 11,001 SNPs passing the stacks filters with minor allele frequency >0.05 and representation in at least 70% of all samples. Mean depth of coverage per sample for filtered SNPs is 20.1 (range: 6.2–49.9) (Fig. S5; Table S1), and mean proportion of loci with missing data per sample is 0.22 (range: 0.04–0.73) (Table S1). Similarly, we found 11,527 SNPs meeting those criteria considering only samples within the island of Hawaii.

Histograms of the fraction of reads supporting each allele for 40 individuals that have at least 1,000 loci with at least 60 × coverage demonstrate that heterozygotes are primarily called from situations in which support for each allele is approximately 50:50 (0.5) (Fig. S6). In many cases, the tails of the histogram extend to 0.25 and 0.75, but only in a few cases are subtle peaks suggested. As a result, we believe that koa may be allotetraploid (amphidiploid) and our pipeline is separating homeologs, meaning that there is likely very little bias in the genotype calls or downstream analyses.

### 3.2 Genetic diversity and structure

**Figure 1:**
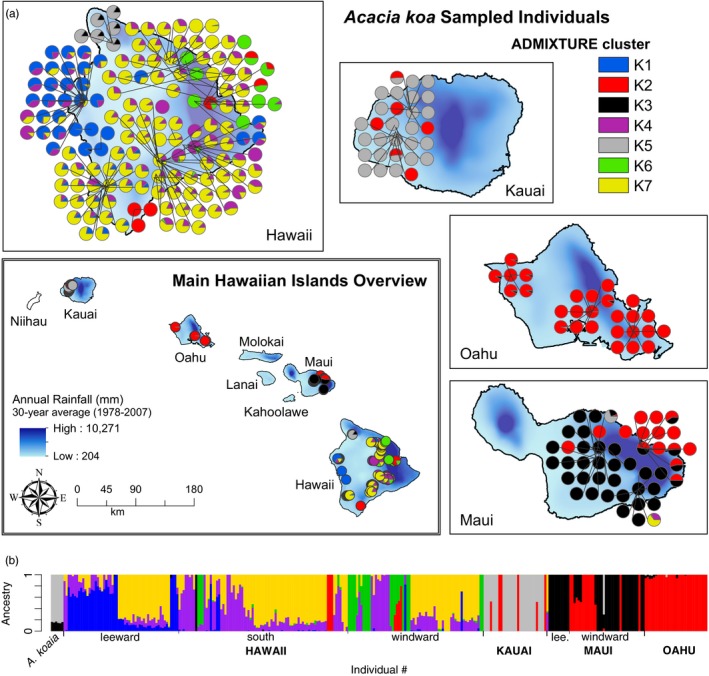
(a) Map depicting individual assignments to seven genetic clusters inferred from admixture against a rainfall gradient as the background map color. (b) Individuals (vertical bars) colored by proportion assignment to each genetic cluster

**Figure 2:**
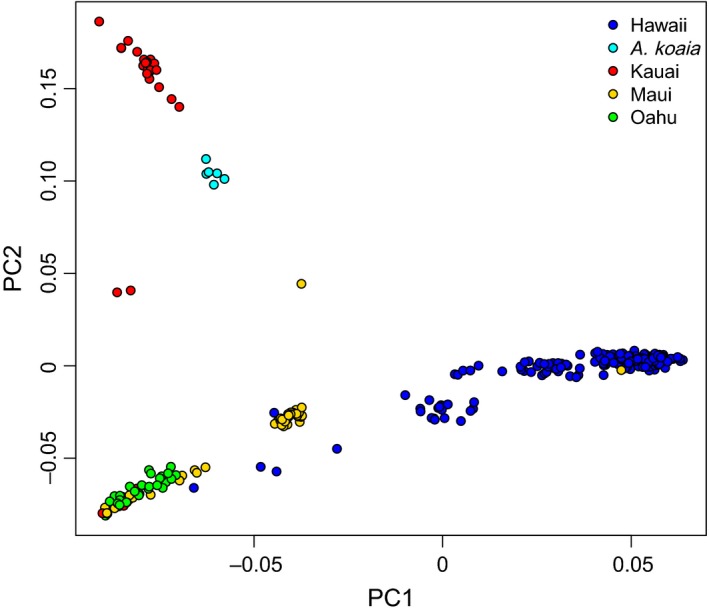
Principal components analysis of 11,001 SNPs across 305 samples of *Acacia koa* and 6 *A. koaia*

Genetic diversity is moderate: 0.29 < *H *<* *0.33 and 0.0016 < π < 0.0018 (Table 1). *F*_ST_ values among islands and among admixture clusters are moderate to high for a tree species: 0.05 < *F*_ST_ < 0.19 (Table 2). These relatively high *F*_ST_ values might represent further support for our argument that koa is allotetraploid. If koa were autotetraploid and analyzed as a diploid, then there would be an excess of heterozygotes due to binning all heterozygotes, which would depress *F*_IS_ and *F*_IT_ to negative values and consequently reduce or “cancel out” all but the largest *F*_ST_ values. The “optimal” number of clusters as inferred from admixture is 7 when considering all samples and 5 when considering only the island of Hawaii (Figure 1). The latter is essentially the same as a subset of those inferred across all samples, and thus, only the overall result is shown. Kauai and Oahu are dominated by one cluster each; Maui is a mix of two clusters, one of which is the same as in Oahu; and Hawaii contains five of its own clusters with some geographic structure (e.g., windward versus leeward) and individuals in various levels of admixture. *A. koaia* (from Hawaii) may be the product of admixture from individuals from Maui and Kauai. Cluster membership is generally not significantly correlated with the amount of missing data nor coverage after accounting for multiple testing (−0.15 < *r *< 0.11, *p *> 0.05), except for cluster K4 which is weakly but significantly associated with missing data percentage (*r *= 0.23, *p *= 0.0004). PCA reveals some notable differences among islands and shows similar patterns to those from admixture (Figure 2). The first principal components axis separates Hawaii from other islands, shows some structure within Hawaii, and shows that some samples in Maui are distinct from those on Oahu. The second axis separates the samples in Kauai and suggests that *A. koaia* is distinct but genetically similar to Kauaian samples of *A. koa*. In both the ordination and the admixture plots, a few “stray” individuals appear to cluster with the “wrong” island, suggesting they may have been moved or arrived there recently.

**Table 1:** Mean expected heterozygosity (*H*), nucleotide diversity (π), and their standard errors (*SE*) estimated for each island

Island	*H*	*SE*	π	*SE*
Hawaii	0.33	0.0014	0.0018	0
Kauai	0.30	0.0013	0.0016	0
Maui	0.29	0.0015	0.0017	0
Oahu	0.29	0.0015	0.0017	0

**Table 2:** Pairwise *F*_ST_ among islands and among genetic clusters inferred from admixture

Island	Hawaii	Oahu	Kauai	Maui
Hawaii	–			
Oahu	0.12	–		
Kauai	0.14	0.14	–	
Maui	0.08	0.05	0.13	–

Cluster	K1	K2	K3	K4	K5	K6	K7
K1	–						
K2	0.12	–					
K3	0.10	0.08	–				
K4	0.09	0.15	0.13	–			
K5	0.16	0.16	0.15	0.19	–		
K6	0.10	0.13	0.10	0.11	0.18	–	
K7	0.08	0.14	0.11	0.05	0.16	0.11	–

### 3.3 Environmental and spatial associations with genetic variation

**Figure 3:**
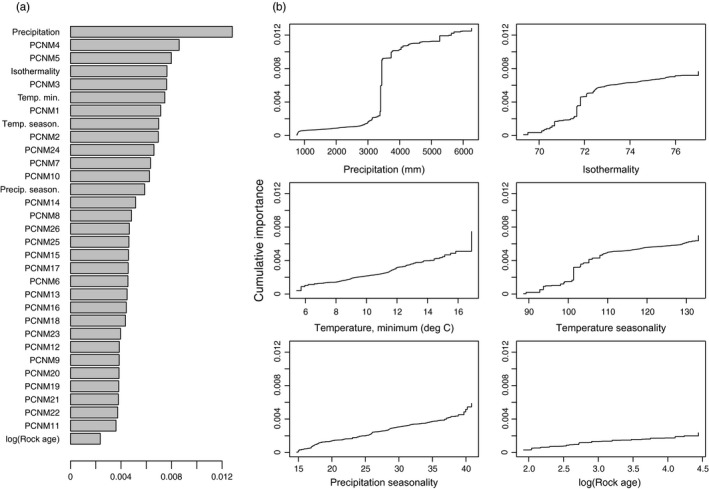
(a) *R* ^2^‐weighted importance of environmental and spatial variables for explaining genetic gradients from gradient forest analysis. (b) Cumulative importance of allelic change along six environmental gradients

GF analysis indicates that mean annual rainfall is the single most important predictor among all environmental and spatial variables considered; isothermality, minimum temperature, temperature seasonality, and rainfall seasonality have moderate importance; and log of the rock substrate age has little importance (Figure 3a). Summing importances of all PCNMs, the results suggest that spatial variables explain 84% of variation and environmental variables explain 16%, although these estimates may vary when considering different numbers of spatial and environmental variables. Allelic composition changes sharply between 3,000 and 4,000 mm/yr rainfall, whereas changes along other environmental variables occur gradually or with modest step‐changes, if at all (Figure 3b).

**Figure 4:**
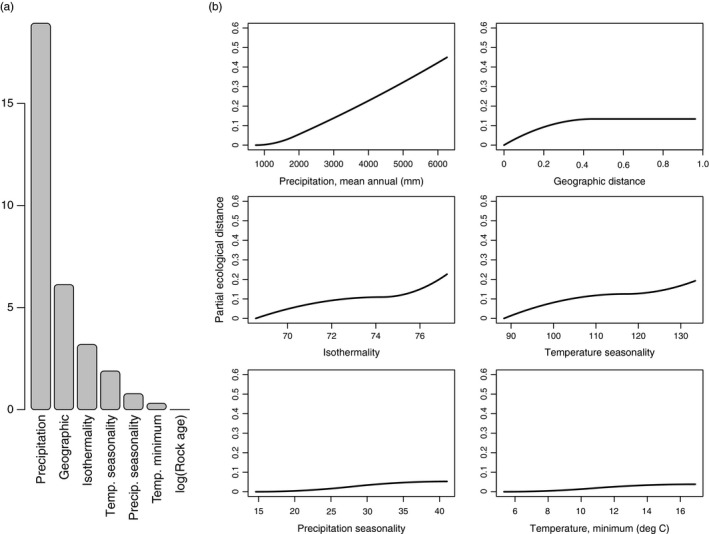
(a) Variable importance and (b) *I*‐splines showing changes genetic distance along environmental gradients as modeled by generalized dissimilarity modeling. Splines reaching higher values have higher importance. *I*‐spline plot for rock substrate age is not shown because all coefficients equal zero

Geographic and environmental distances for the variables considered with GDM explain 32.9% of allelic variation (deviance). Annual rainfall is by far the most important predictor, whereas there is almost no contribution of minimum temperature or rock substrate age, and all other variables contribute modestly (Figure 4). Rainfall varies more linearly in the GDM results compared to the GF results, and unlike the GF results, environmental variables explain more variation (80%) than the spatial variable (20%).

**Figure 5:**
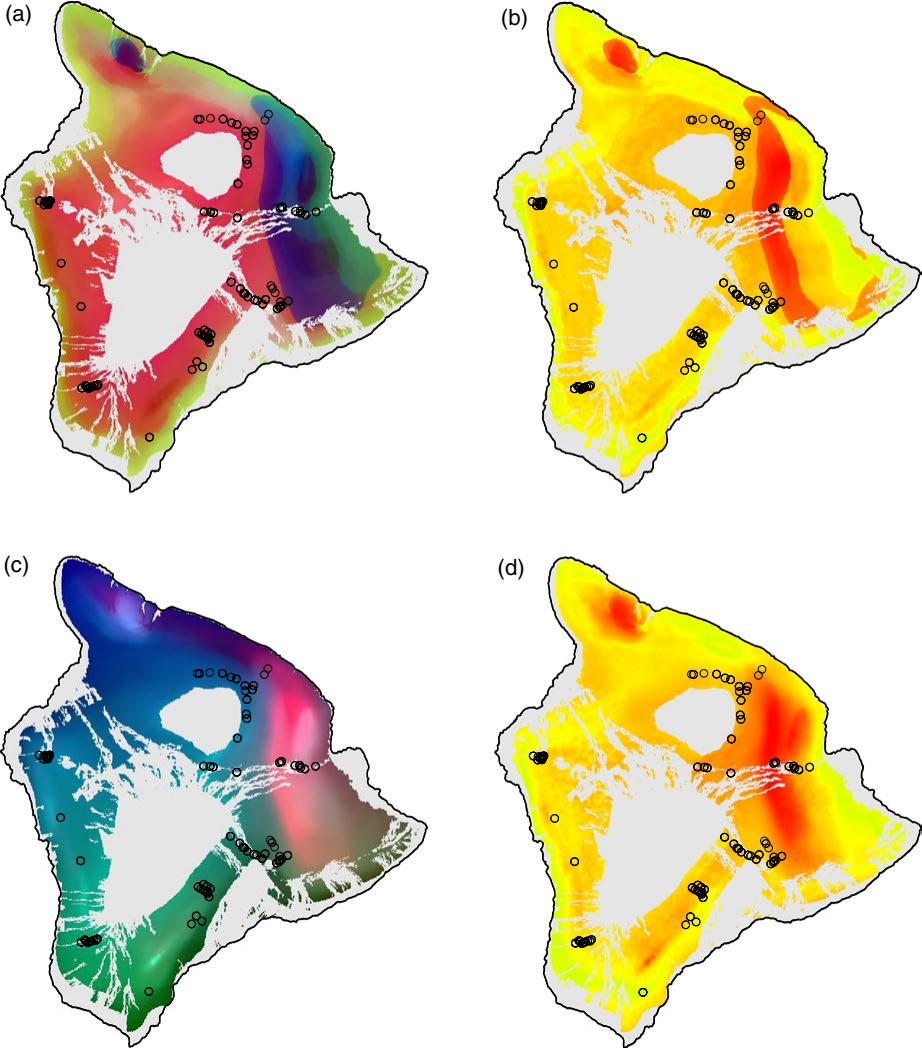
Current landscape patterns of allelic composition as predicted from transformed environmental variables after adjusting for spatial variation from (a) gradient forest and (c) general dissimilarity models. Within each of these panels, similar colors represent similar expected genetic compositions (colors are not comparable between these panels). Under future climate, “genetic offset” depicting areas that will be most discordant genetically are shown for (b) gradient forest and (d) general dissimilarity models. Higher offset (more red) areas can be interpreted as regions of higher vulnerability to predicted future change. Landscape is masked by the current predicted distribution of *A. koa*/*A*. *koaia* (Price et al., 2012)

Mapped projections of GDM and GF results onto the landscape are generally concordant and show that eastern part of the island of Hawaii, which is the windward side where rainfall is high, is genetically different from the rest of island (Figure 5). This eastern area also exhibits the highest genetic offset under our future climate change scenario, owing to predicted decreases in rainfall in this region.

## 4 DISCUSSION

### 4.1 Biogeography

Genetic structure among islands is relatively strong and complicated, suggestive of isolation with periodic dispersal. Evidence of isolation is seen in the PCA and admixture results that show distinct clusters that are unique to each island (Figures 1 and 2). Most individuals on the small islands are genetically pure, whereas the big island of Hawaii is composed of several highly admixed clusters (Figure 1) or relatively continuous variation (Figure 2) specific to that island. Nonetheless, dispersal appears to be an important force structuring variation among islands. For example, the Oahu genetic cluster is found prominently on Maui near its northeastern coast, where a few individuals are admixed (possible F1s), as well as in a few pure individuals on Kauai and Hawaii (Figure 1). These dispersal events are likely recent, given the general lack of admixture or apparent backcrosses. For example, they are not consistent with a long history of pollen dispersal with prevailing winds from northeast to southwest (e.g., Gugger & Cavender‐Bares, 2013). Given the cultural and economic importance of the species, along with active restoration programs, it is likely that the observed recent dispersal events relate to human activities, although we cannot rule out other mechanisms. Hints of deeper biogeographic history are suggested in the PCA (Figure 2) and pairwise *F*_ST_ estimates (Table 2), which show that populations on Oahu and Maui are most similar. Hierarchical clustering analyses of genetic variation presented in Dudley et al. (2017) suggest that these populations are in turn most similar to *A. koa* on Kauai and least similar to those on Hawaii, also consistent with our findings by island and cluster (Table 2). Without rooting these relationships, it is hard to test whether the dispersal and colonization history follows a stepping‐stone pattern from oldest (Kauai) to youngest (Hawaii) island.

Interestingly, *A. koaia* on Hawaii falls within the range of genetic variation for *A. koa* but forms a distinct group that is most similar to *A. koa* populations on Kauai without signs of admixture with populations from the island of Hawaii (Figure 2). Therefore, *A. koaia* may be a drought‐adapted ecotype formed following dispersal to Hawaii from Kauai, possibly through stabilized admixture (“hybrid swarm”) of Kauaian populations with a smaller fraction of Mauian populations (Figure 1). This scenario also suggests that Kauaian populations may not readily interbreed with populations of *A. koa* from the island of Hawaii, facilitating ecotypic divergence on Hawaii. If *A. koaia* continues to be considered a different species or subspecies on the basis of morphology, ecology, and genetics, our genetic data suggest that populations on Kauai might also represent a cryptic species. Indeed, it might be argued that populations on each island can be considered a different subspecies according to the observed genetic clustering with limited admixture as well as previously reported isozyme and genetically based morphological differences among the island of Hawaii and the other islands (Conkle, 1996; Daehler et al., 1999; Shi, 2003; Sun, 1996). However, further morphological and ecological investigations are warranted.

### 4.2 Rainfall structures diversity

Within the island of Hawaii, variation is partially structured by geographic factors but may be mostly driven by rainfall gradients. Both GF and GDM analyses indicate that rainfall is by far the most important variable associated with genetic variation (Figures 3 and 4). The sharp changes in genetic composition from the wet, windward (eastern) side of the island to the dry, leeward (western) side are striking in both landscape projections. The primary disagreements among the methods are the relative role of spatial and environmental variables and whether the relationship with rainfall is approximately linear (GDM) versus a steep step function (GF), both of which can be attributed to differences in statistical approach and sensitivity, as well as the number of spatial versus environmental variables considered in each model.

Genomewide associations with environmental variables can result from geographic, demographic, or selective forces (Wang & Bradburd, 2014). We do not believe geographic forces alone, such as physical barriers on the island of Hawaii, explain this result. The topographic features of the island of Hawaii where genetic change is steepest are not likely to block dispersal, and in fact, pollen is likely to readily disperse from the windward to leeward side of the island, promoting homogenization along this axis for which we observe the sharpest differences. Furthermore, individuals at the southern, wet end of Mauna Loa Road (Table S1) are not physically blocked from individuals located further upslope along the drier stretch of road, but the southern samples are genetically more similar to windward populations (Figure 1). Instead, other demographic and selective forces may be at play. For example, steep environmental differences over small spatial scales can lead to differences in flowering phenology among nearby individuals, biasing mating patterns toward those with similar environmentally controlled phenology leading to, or magnifying, genotype–environment associations (Soularue & Kremer, 2014). Moreover, environmental gradients might influence the behavior or distributions of key insect dispersers of koa pollen and/or animal dispersers of its seeds. Another explanation for the observed associations is local or clinal adaptation to rainfall regimes (e.g., water stress). Strong genotype–environment correlations after accounting for spatial variables, as we observe, have been attributed to local adaptation (e.g., Manel et al., 2010). Furthermore, it is clear that water stress plays an important role in ecotypic differentiation between *A. koa* and *A. koaia* on Hawaii (Baker et al., 2009) and *A. koa* populations exhibit genetically based differences in water use efficiency along elevation gradients on the island (Ares, Fownes, & Sun, 2000), suggesting the importance of adaptation to water stress more generally for this genus in this setting. In addition, a number of koa provenance tests more broadly demonstrate local adaptation of koa population to home environments among islands and offer preliminary evidence of differences among populations within islands based on general growth and morphological quantitative traits (Conrad, Fulii, & Ikawa, 1995; Daehler et al., 1999; Shi, 2003; Sun, 1996). Explanations based on demographic and selective mechanisms are not mutually exclusive, as natural selection and assortative mating by phenology can serve to reinforce each other (Andrew, Ostevik, Ebert, & Rieseberg, 2012; Via, Bouck, & Skillman, 2000), and patterns of neutral and adaptive genetic variation can be correlated for a number of reasons (e.g., Gugger, Cokus, & Sork, 2016; Sork et al., 2016; Wang & Bradburd, 2014). Thus, our findings are consistent with isolation‐by‐environment or isolation‐by‐adaptation models (Nosil, Funk, & Ortiz‐Barrientos, 2009; Orsini, Vanoverbeke, Swillen, Mergeay, & De Meester, 2013; Wang & Bradburd, 2014).

### 4.3 Risk of genetic offset

Because rainfall appears to be so important in shaping variation, it is unsurprising that predictions of genetic offset, based on a future climate scenario, suggest that the most vulnerable populations are along the edge of the windward–leeward transition zone (Figure 5) where rainfall is expected to decline in the future according to the model we selected (Figs. S3 and S4). Regardless of whether the associations are driven by demographic or selective forces, we expect that changing environment will lead to the greatest genetic changes from today's genetic variation to expected future variation. To the extent that these relationships are adaptive, we can assign vulnerabilities to projected change where population may be most maladapted to future environments. Further work integrating population genomic and quantitative genetic approaches is needed to break down the contribution of demographic and adaptive forces, and monitoring of koa populations might provide empirical data to validate the model projections of vulnerability.

### 4.4 Management implications

We observed strong genetic structure among islands, clusters, and ecotypes, and understanding these population differences can inform land management decisions. For example, preliminary seed transfer guidelines follow ecological zones within islands (Dudley et al., 2017), but these zones might be refined considering the patterns observed here. More sampling on each island, particularly the smaller islands, would help to better define the composition of different clusters on islands with mixed composition. The strong genetic structure also offers an opportunity to further explore local adaptation among groups and exploit these, and crosses, for agroforestry.

We have found that rainfall is a major force shaping variation within the island of Hawaii. Whether this pattern is driven by demographic forces related to rainfall (e.g., via phenology) or due to natural selection and local adaptation, we expect that climate change will exert pressure on populations in areas where rainfall regimes will change most. These vulnerable areas might be candidates to consider moving genotypes (Aitken & Whitlock, 2013) from other regions that are “preadapted” to the expected future conditions (e.g., moving genotypes from dry regions to vulnerable areas where rainfall will decline following associations observed in GDM and GF). More generally, the strong genetic differences within the island of Hawaii along gradients can guide plantings even when considering only present patterns. For example, previously proposed ecologically based seed zones (Dudley et al., 2017) might be refined to account for the axes of variation observed here.

We show the utility of predictive, nonlinear association modeling for identifying vulnerable and resilient populations. However, our findings should be modified as better downscaled climate data are developed to account for unique aspects of Hawaii that may not be well accounted for in global circulation models. To the extent that the models used here are accurate, we identify specific regions for concern. Overall, this approach can be readily applied to guide planting strategies for any species of conservation or economic interest utilizing genomic (e.g., RAD‐Seq/GBS) data.

## DATA ARCHIVING STATEMENT

Illumina sequence read data are available through NCBI BioProject PRJNA392301, and final SNP data files are available through Dryad https://doi.org/10.5061/dryad.c014p.

## Supporting information


** **

** **

** **
