# Supplementary material for: Applying landscape genomic tools to forest management and restoration of Hawaiian koa (Acacia koa) in a changing environment
Source: Evol Appl. 2017 Sep 6;11(2):231–42. doi: 10.1111/eva.12534 (PMC5775490; doi:10.1111/eva.12534)
Supplement: Supplementary file 1 [file EVA-11-231-s001.docx]

*Evolutionary Applications*

**SUPPORTING INFORMATION**

**Applying landscape genomic tools to forest management and restoration**

**of Hawaiian koa (*Acacia koa*) in a changing environment**

Paul F. Gugger, Christina T. Liang, Victoria L. Sork, Paul Hodgskiss, and Jessica W. Wright

Tables S1–S4

Figures S1–S6

**Table S1.** Name, locality, species, coordinates, mean depth of coverage per sample, proportion missing data per sample, environmental variables, and other information for each sample

(see TableS1.xlsx)

**Table S2.** Inline genotype-by-sequencing barcodes used for multiplexed Illumina sequencing

| **Barcodes** | |
| --- | --- |
| AACCGAACT | GATCGTTCAGA |
| AAGACAGGC | GCAGGAAAAGTT |
| AAGGTCGAT | GCCTTAACGCCT |
| AATAGCTCC | GCGAAAATATGC |
| ACCAGGCGT | GCTCTATGAAAC |
| ACCTATCAC | GCTGCATTAATT |
| AGAGTAGGAT | GCTTGATTGGAT |
| AGGCTCCTAC | GGCGTCTACGGA |
| ATAAGCTGTA | GGTCCGAACTTC |
| ATTCAGGAAC | GTAATGAATTCA |
| ATTGGGGTGT | GTCTATAGCGGA |
| CAACTGTATT | TATCTAACCGAGA |
| CAATCTAATA | TATTGACGACTAC |
| CAGCGTACAT | TCATCCCATGGGT |
| CCATATGCGA | TCCGGCCGGATAT |
| CCGACTTCTC | TCGTACGCGGAGA |
| CGATTATCGTA | TCTACCGTGTGGT |
| CGGCACCAGCT | TGAGAGCTGTGGA |
| CGTCGCGCGGT | TGCGCGGATTGGT |
| CTCAGCTTCCA | TGCTAGTGAGGGT |
| CTCGCGCCAGT | TTACTTAGGCCAT |
| CTGCCGCTCTA | TTAGCTATCGGGA |
| CTTAAGGTTGT | TTATGTCTCAGTC |
| GATATTGGCTA | TTCGATGGTACGT |

**Table S3.** Summary statistics (expected heterozygosity and nucleotide diversity) and their standard errors resulting from repeated runs of Stacks with different parameter values for samples with technical replicates

| **Parameters tested*** | | | | | | |  |  |  |  |  |
| --- | --- | --- | --- | --- | --- | --- | --- | --- | --- | --- | --- |
| **-m** | | **-M** | | **-n** | **--max_locus_stacks** | **--bound_high** | **Polymorphic Sites** | ***H*** | **SE** | **π** | **SE** |
| 3 | 2 | | 2 | | 3 | 0.05 | 3424 | 0.28 | 0.003 | 0.0017 | 0 |
| 4 | 2 | | 2 | | 3 | 0.05 | 1326 | 0.25 | 0.004 | 0.0013 | 0 |
| 5 | 2 | | 2 | | 3 | 0.05 | 535 | 0.22 | 0.006 | 0.001 | 0 |
| 3 | 3 | | 3 | | 3 | 0.05 | 2916 | 0.27 | 0.003 | 0.0017 | 0 |
| 3 | 2 | | 2 | | 4 | 0.05 | 3427 | 0.28 | 0.003 | 0.0017 | 0 |
| 3 | 2 | | 2 | | 5 | 0.05 | 3429 | 0.28 | 0.003 | 0.0017 | 0 |
| 3 | 1 | | 1 | | 3 | 0.05 | 3547 | 0.28 | 0.003 | 0.0013 | 0 |
| 4 | 1 | | 1 | | 3 | 0.05 | 1457 | 0.26 | 0.004 | 0.0011 | 0 |
| 3 | 2 | | 2 | | 3 | 0.1 | 3217 | 0.30 | 0.003 | 0.0016 | 0 |
| 4 | 2 | | 2 | | 3 | 0.1 | 1201 | 0.26 | 0.004 | 0.0012 | 0 |

*Underlined values are those that were selected for the primary data analyses presented in this paper.

**Table S4.** Pairwise correlations among coordinates and environmental variables considered for this study

(See TableS4.xlsx)


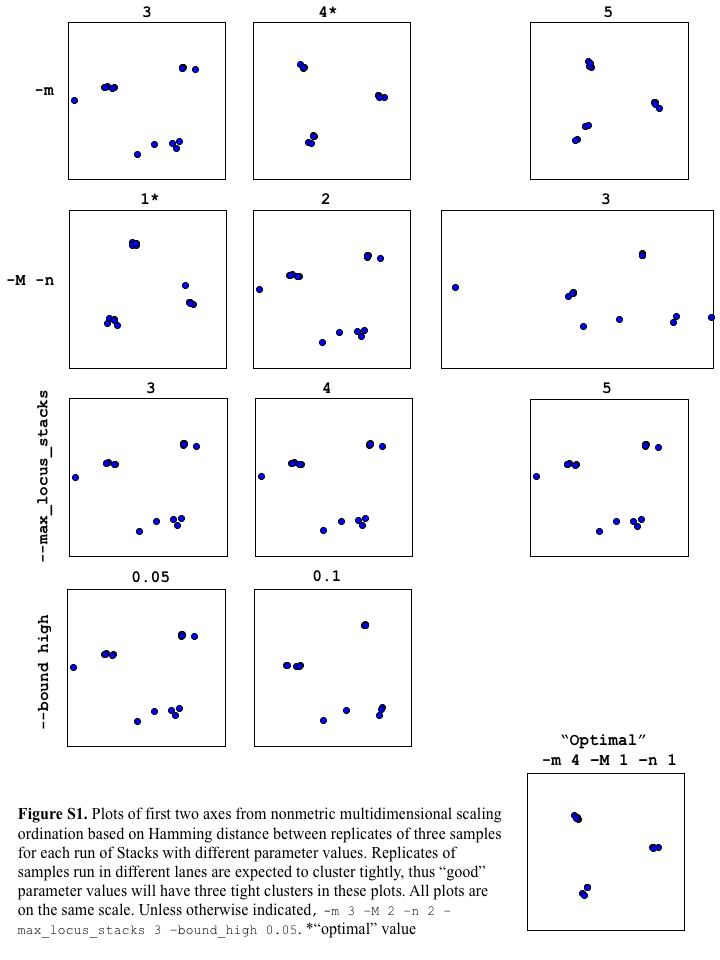


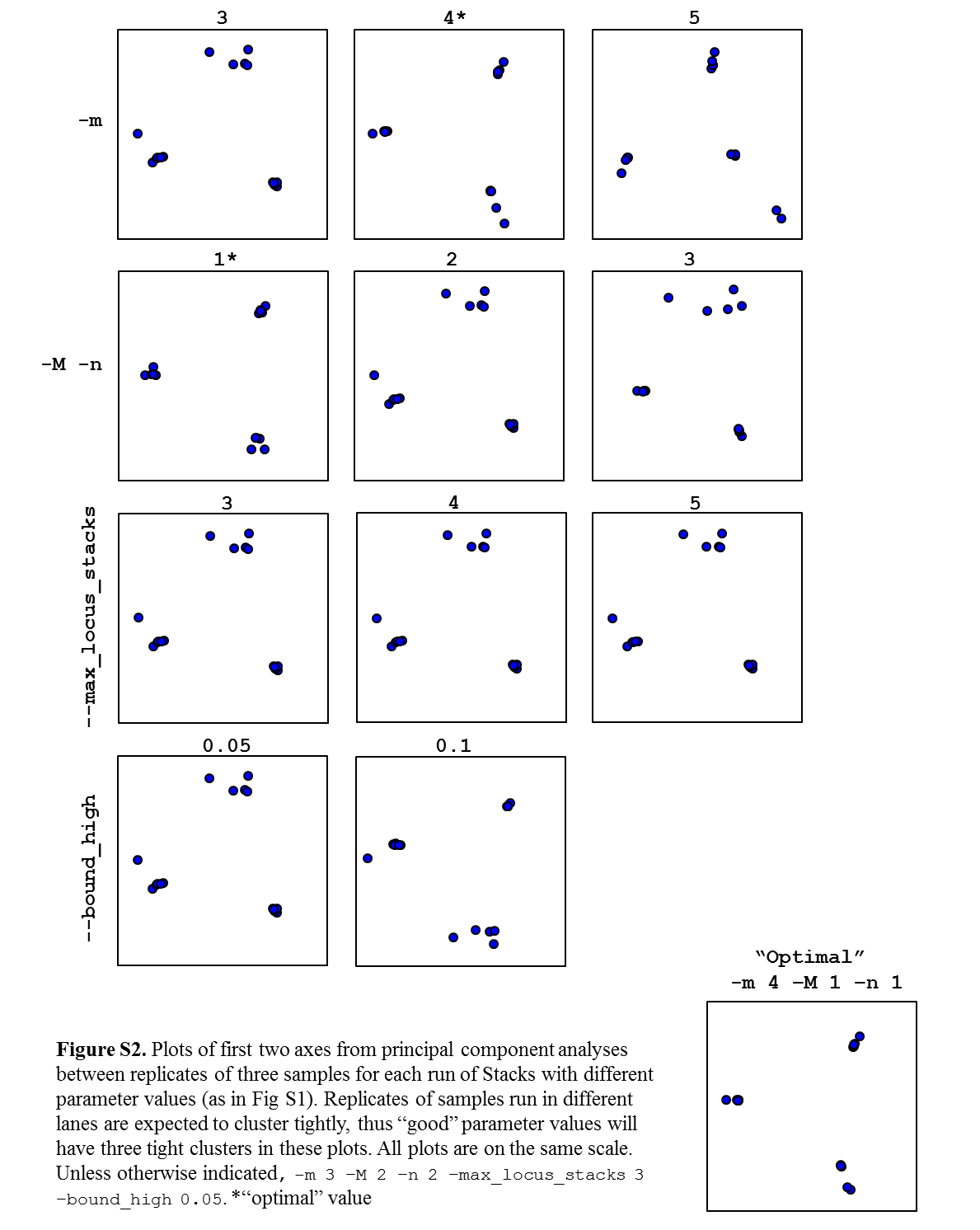


Latitude


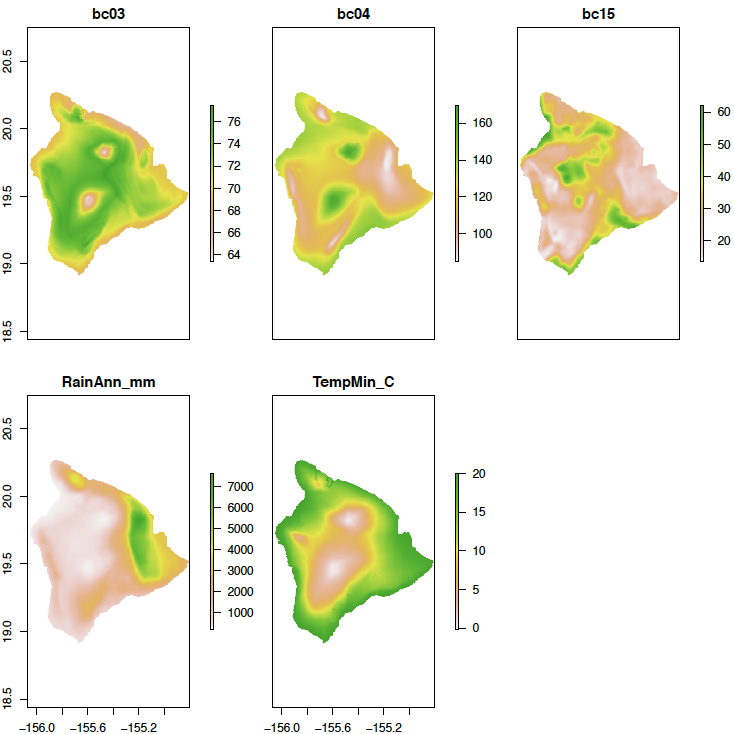


Longitude

**Figure S3**. Mapped variation in current climate variables used in this study. bc03 = isothermality, bc04 = temperature seasonality, bc15 = precipitation seasonality, RainAnn_mm = mean annual precipitation in mm, TempMin_C = and mean minimum temperature in °C.


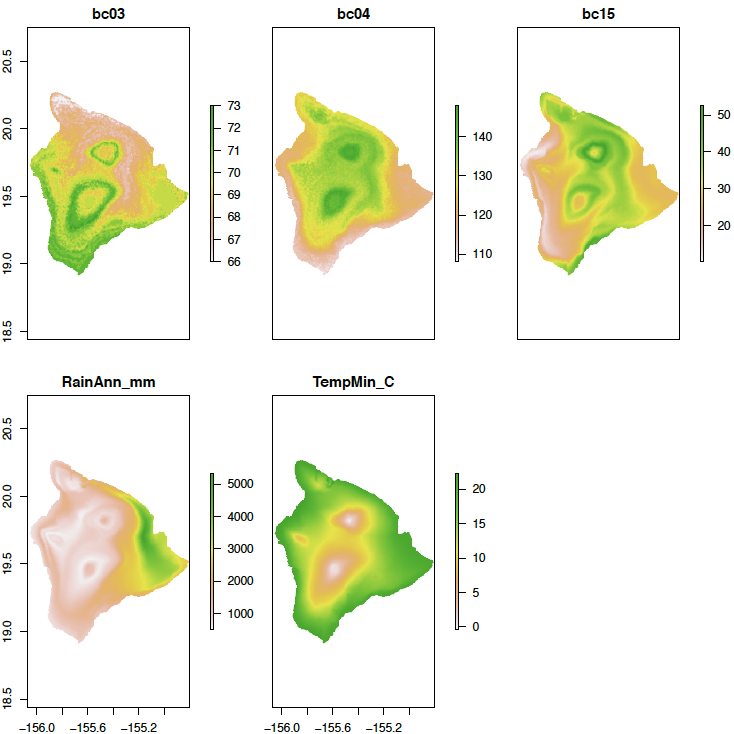


Latitude

Longitude

**Figure S4**. Mapped variation in future climate variables from the IPCC5 CMIP5 data set with CESM1-CAM5-1-FV2 global circulation model under the Representative Concentration Pathway 4.5 greenhouse gas emissions scenario for the year 2070. bc03 = isothermality, bc04 = temperature seasonality, bc15 = precipitation seasonality, RainAnn_mm = mean annual precipitation in mm, TempMin_C = and mean minimum temperature in °C.


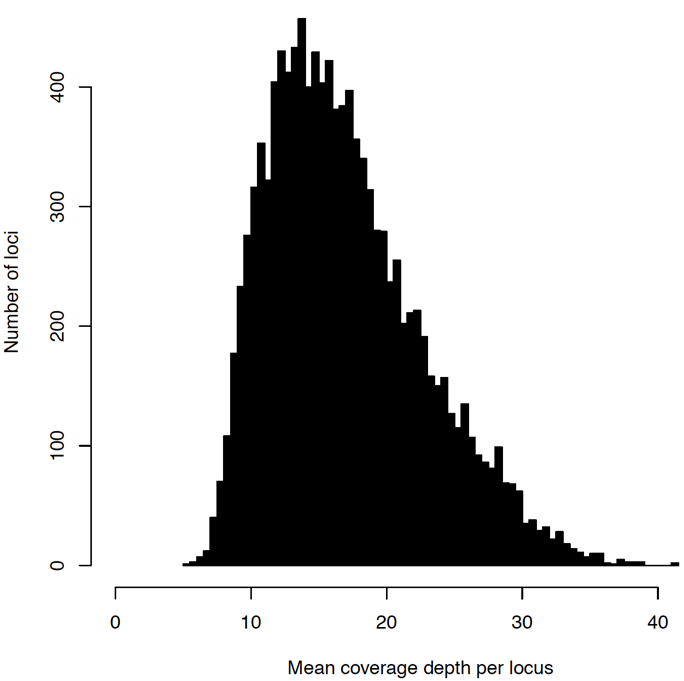

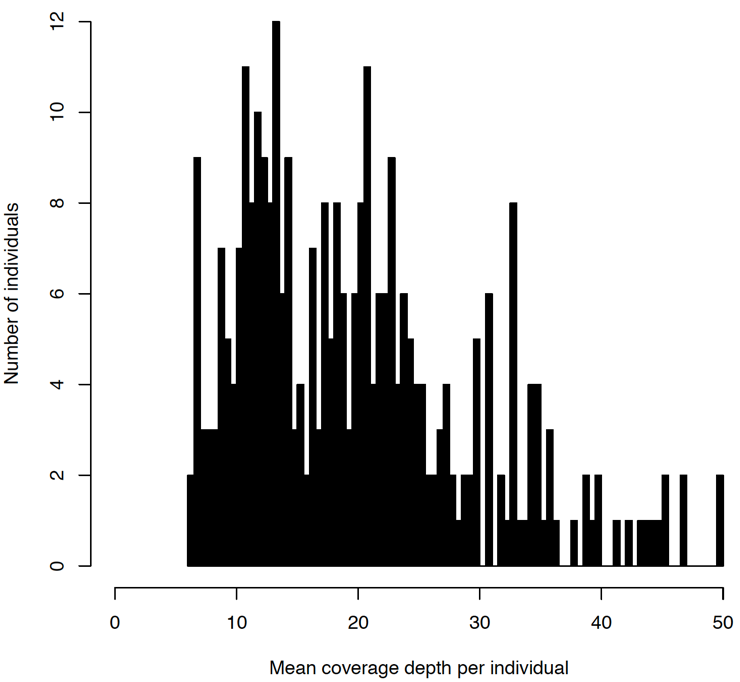


**(a)**

**(b)**

**Figure S5.** Histograms of **(a)** mean coverage depth per locus and **(b)** mean coverage depth per individual (missing data excluded).

**Figure S6.** Histograms of the fraction of reads supporting each allele for 40 individuals that had at least 1000 loci with at least 60× coverage.
